# Dementia Risk Factors Are Undiagnosed and/or Uncontrolled in a Latino and Black Aging Cohort

**DOI:** 10.1002/alz.092985

**Published:** 2025-01-09

**Authors:** Christian Lachner, Yoav D Piura, Paula Aduen, Minerva M. Carrasquillo, Richard O White, Neill R. Graff‐Radford, Gregory S Day

**Affiliations:** ^1^ Mayo Clinic, Jacksonville, FL USA

## Abstract

**Background:**

The prevalence of dementia in populations that are underrepresented in research is projected to increase with population aging. Latino and Black individuals, the largest underrepresented populations in the US, experience a higher dementia burden than non‐Hispanic White individuals, which is not explained by ethnoracial biological factors. Disparities in structural and social determinants of health may contribute to the preponderance of medical conditions known to increase dementia risk, including potentially modifiable factors in Latino and Black communities. To assess the scope of this problem, we measured the frequency of common modifiable dementia risk factors in community‐dwelling, cognitively normal, Latino and Black adults.

**Method:**

Cognitively normal community‐dwelling Latino and Black adults, ≥45‐years‐old, were enrolled within our longitudinal study of memory and aging at Mayo Clinic in Florida (Jacksonville, FL) and completed a baseline assessment between 3/2022 and 10/2023. Demographics, and self‐reported race/ethnicity and history of dementia risk factors were recorded. Dementia risk factors were objectively sampled through clinical interview (depression: Geriatric Depression Scale ≥5), examination (hypertension: BP >130/80 mmHg; obesity: BMI≥30), and serum analyses (hyperlipidemia: total cholesterol<200mg/dL, HDL>50mg/dL, LDL<100mg/dL, Triglycerides<150mg/dL; diabetes: HbA1C >5.6%, vitamin B12 deficiency: <350 ng/dL).

**Result:**

86 participants (43 Latino, 43 Black), 65.8 (8.8) years‐old, female (62.8%), with 16 (2.4) years of education were assessed. Dementia risk factors were frequent in Latino and Black participants, including hypertension (63%, 90%; p = 0.005), hyperlipidemia (93%, 89%; p = 0.702), obesity (33%, 44%; p = 0.370), diabetes (60%, 47%; p = 0.370), vitamin B12 deficiency (21%, 21%; p = 0.989), and depression (5%, 24%; p = 0.781). These risk factors were unrecognized or suboptimally managed in a substantial proportion of Latino and Black participants: hypertension (56%, 73%; p = 0.170), hyperlipidemia (80%, 73%; p = 0.591), diabetes (57%, 47%; p = 0.502), vitamin B12 deficiency (21%, 21%; p = 0.789), and depression (5%, 3%; p>0.99).

**Conclusion:**

Potentially modifiable dementia risk factors were detected in the majority of community‐dwelling Latino and Black cognitively normal older‐adults and were frequently unrecognized or suboptimally managed. Proactive screening for known dementia risk factors may provide opportunities for targeted interventions to improve brain health and decrease dementia disparities in underrepresented populations.

